# 1-[1-(4-Nitro­phen­yl)ethyl­idene]thio­semicarbazide

**DOI:** 10.1107/S1600536808025105

**Published:** 2008-08-09

**Authors:** Jian-Gang Wang, Fang-Fang Jian, Yu-Feng Ding

**Affiliations:** a Microscale Science Institute, Bioengineering School, Weifang University, Weifang 261061, People’s Republic of China; b Microscale Science Institute, Weifang University, Weifang 261061, People’s Republic of China; c Number Seven Middle School, Weifang 261061, People’s Republic of China

## Abstract

The title compound, C_9_H_10_N_4_O_2_S, was prepared by the reaction of 1-(4-nitro­phen­yl)ethanone and thio­semicarbazide in ethanol at 367 K. There are weak inter­molecular N—H⋯S and N—H⋯O hydrogen-bonding inter­actions in the crystal structure involving the amine and nitrile groups, respectively, as donors.

## Related literature

For related literature, see: Jian *et al.* (2006 ▶); Qin *et al.* (2006 ▶); Rozwadowski *et al.* (1999 ▶).
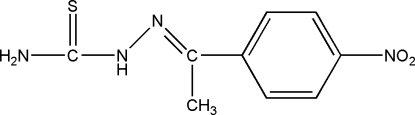


## Experimental

### 

#### Crystal data

C_9_H_10_N_4_O_2_S
*M* *_r_* = 238.27
Triclinic, 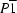

*a* = 7.4450 (15) Å
*b* = 9.3180 (19) Å
*c* = 9.4050 (19) Å
α = 62.08 (3)°
β = 76.41 (3)°
γ = 69.02 (3)°
*V* = 536.5 (3) Å^3^
*Z* = 2
Mo *K*α radiation
μ = 0.29 mm^−1^
*T* = 293 (2) K
0.20 × 0.15 × 0.10 mm

#### Data collection

Bruker SMART CCD area-detector diffractometer
Absorption correction: none
2493 measured reflections
2307 independent reflections
1776 reflections with *I* > 2σ(*I*)
*R*_int_ = 0.026

#### Refinement

*R*[*F* ^2^ > 2σ(*F* ^2^)] = 0.046
*wR*(*F* ^2^) = 0.140
*S* = 1.08
2307 reflections
145 parameters
H-atom parameters constrained
Δρ_max_ = 0.39 e Å^−3^
Δρ_min_ = −0.38 e Å^−3^

### 

Data collection: *SMART* (Bruker, 1997 ▶); cell refinement: *SAINT* (Bruker, 1997 ▶); data reduction: *SAINT*; program(s) used to solve structure: *SHELXS97* (Sheldrick, 2008 ▶); program(s) used to refine structure: *SHELXL97* (Sheldrick, 2008 ▶); molecular graphics: *SHELXTL* (Sheldrick, 2008 ▶); software used to prepare material for publication: *SHELXTL*.

## Supplementary Material

Crystal structure: contains datablocks I, global. DOI: 10.1107/S1600536808025105/at2598sup1.cif

Structure factors: contains datablocks I. DOI: 10.1107/S1600536808025105/at2598Isup2.hkl

Additional supplementary materials:  crystallographic information; 3D view; checkCIF report

## Figures and Tables

**Table 1:** Hydrogen-bond geometry (Å, °)

*D*—H⋯*A*	*D*—H	H⋯*A*	*D*⋯*A*	*D*—H⋯*A*
N3—H3*A*⋯S1^i^	0.86	2.74	3.581 (2)	166
N4—H4*A*⋯O1^ii^	0.86	2.35	3.101 (3)	146
N4—H4*B*⋯O2^iii^	0.86	2.29	3.133 (3)	166

Symmetry codes: (i) 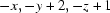; (ii) 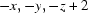; (iii) 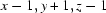.

**Fig. 1.**
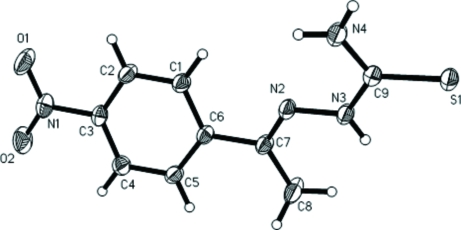
The structure of the title compound showing 30% probability displacement ellipsoids and the atom-numbering scheme.

## supplementary crystallographic information

### Comment

Schiff bases have been used extensively as ligands in the field of coordination chemistry (Jian *et al.*, 2006). Schiff bases show biochemical and pharmacological applications. The growing interest in Schiff bases lately is also due to their ability to form intramolecular hydrogen bonds by electron coupling between acid-base centers (Rozwadowski *et al.*,1999). The title compound (I), was synthesized and we report here its crystal structure

In the crystal structure of (I) (Fig. 1). The C6–C9/N2/N3/S1 plane makes a dihedral angle of 19.78 (127)° with the benzene ring (C1—C6). The C═N bond length [1.281 (3) Å] and C═S bond length [1.685 (2) Å] are in agreement with those observed before (Jian *et al.*, 2006; Qin *et al.*, 2006). There are intermolecular N–H···S and N–H···O hydrogen-bond interactions to stabilize the crystal structure (Table 1).

### Experimental

A mixture of hydrochloric acid 0.6 mL (0.02 mol) and thiosemicarbazide 1.8 g (0.02 mol) was stirred with ethanol (50 mL) at 293 K for 2 h, then add 1-(4-nitrophenyl)ethanone 3.3 g (0.02 mol), then afford the title compound [4.17 g, yield: 87.6%]. Single crystals suitable for X-ray measurements were obtained by recrystallization from acetone and ethanol(1:1) at room temperature.

### Refinement

H atoms were fixed geometrically and allowed to ride on their attached atoms, with C—H and N—H distances of 0.93–0.96 and 0.86 Å, and with *U*_iso_=1.2 or 1.5*U*_eq_.

### Crystal data

**Table:**

C_9_H_10_N_4_O_2_S	*Z* = 2
*M**_r_* = 238.27	*F*_000_ = 248
Triclinic, *P*1	*D*_x_ = 1.475 Mg m^−^^3^
Hall symbol: -P 1	Mo *K*α radiation λ = 0.71073 Å
*a* = 7.4450 (15) Å	Cell parameters from 1776 reflections
*b* = 9.3180 (19) Å	θ = 2.5–27.0º
*c* = 9.4050 (19) Å	µ = 0.29 mm^−^^1^
α = 62.08 (3)º	*T* = 293 (2) K
β = 76.41 (3)º	Block, yellow
γ = 69.02 (3)º	0.20 × 0.15 × 0.10 mm
*V* = 536.5 (3) Å^3^	

### Data collection

**Table:**

Bruker SMART CCD area-detector diffractometer	1776 reflections with *I* > 2σ(*I*)
Radiation source: fine-focus sealed tube	*R*_int_ = 0.026
Monochromator: graphite	θ_max_ = 27.0º
*T* = 293(2) K	θ_min_ = 2.5º
φ and ω scans	*h* = 0→8
Absorption correction: none	*k* = −11→11
2493 measured reflections	*l* = −11→11
2307 independent reflections	

### Refinement

**Table:**

Refinement on *F*^2^	Secondary atom site location: difference Fourier map
Least-squares matrix: full	Hydrogen site location: inferred from neighbouring sites
*R*[*F*^2^ > 2σ(*F*^2^)] = 0.046	H-atom parameters constrained
*wR*(*F*^2^) = 0.140	*w* = 1/[σ^2^(*F*_o_^2^) + (0.0802*P*)^2^ + 0.1605*P*] where *P* = (*F*_o_^2^ + 2*F*_c_^2^)/3
*S* = 1.08	(Δ/σ)_max_ < 0.001
2307 reflections	Δρ_max_ = 0.39 e Å^−^^3^
145 parameters	Δρ_min_ = −0.38 e Å^−^^3^
Primary atom site location: structure-invariant direct methods	Extinction correction: none

### Special details

**Table:**

Geometry. All e.s.d.'s (except the e.s.d. in the dihedral angle between two l.s. planes) are estimated using the full covariance matrix. The cell e.s.d.'s are taken into account individually in the estimation of e.s.d.'s in distances, angles and torsion angles; correlations between e.s.d.'s in cell parameters are only used when they are defined by crystal symmetry. An approximate (isotropic) treatment of cell e.s.d.'s is used for estimating e.s.d.'s involving l.s. planes.
Refinement. Refinement of *F*^2^ against ALL reflections. The weighted *R*-factor *wR* and goodness of fit *S* are based on *F*^2^, conventional *R*-factors *R* are based on *F*, with *F* set to zero for negative *F*^2^. The threshold expression of *F*^2^ > σ(*F*^2^) is used only for calculating *R*-factors(gt) *etc*. and is not relevant to the choice of reflections for refinement. *R*-factors based on *F*^2^ are statistically about twice as large as those based on *F*, and *R*- factors based on ALL data will be even larger.

### Fractional atomic coordinates and isotropic or equivalent isotropic displacement parameters (Å^2^)

**Table:**

	*x*	*y*	*z*	*U*_iso_*/*U*_eq_	
S1	−0.18728 (10)	0.97254 (7)	0.38617 (7)	0.0483 (2)	
O1	0.2761 (3)	−0.3046 (2)	1.2287 (3)	0.0697 (6)	
O2	0.4183 (3)	−0.2729 (2)	1.3830 (2)	0.0680 (6)	
N1	0.3337 (3)	−0.2167 (2)	1.2626 (2)	0.0472 (5)	
N2	0.0574 (3)	0.5445 (2)	0.7336 (2)	0.0373 (4)	
N3	0.0128 (3)	0.7117 (2)	0.6222 (2)	0.0394 (4)	
H3A	0.0744	0.7778	0.6144	0.047*	
N4	−0.2206 (3)	0.6599 (2)	0.5464 (2)	0.0521 (5)	
H4A	−0.1876	0.5573	0.6190	0.063*	
H4B	−0.3121	0.6918	0.4873	0.063*	
C1	0.1766 (3)	0.1958 (3)	0.9090 (3)	0.0392 (5)	
H1A	0.1182	0.2381	0.8145	0.047*	
C2	0.2135 (3)	0.0244 (3)	1.0120 (3)	0.0410 (5)	
H2B	0.1813	−0.0485	0.9871	0.049*	
C3	0.2990 (3)	−0.0353 (2)	1.1521 (3)	0.0364 (5)	
C4	0.3527 (3)	0.0684 (3)	1.1924 (3)	0.0415 (5)	
H4C	0.4109	0.0249	1.2873	0.050*	
C5	0.3167 (3)	0.2400 (3)	1.0864 (3)	0.0397 (5)	
H5A	0.3537	0.3112	1.1100	0.048*	
C6	0.2260 (3)	0.3065 (2)	0.9453 (2)	0.0337 (4)	
C7	0.1848 (3)	0.4909 (2)	0.8317 (3)	0.0371 (5)	
C8	0.2907 (4)	0.5977 (3)	0.8389 (4)	0.0631 (8)	
H8A	0.2478	0.7120	0.7589	0.095*	
H8B	0.4268	0.5521	0.8185	0.095*	
H8C	0.2649	0.5972	0.9441	0.095*	
C9	−0.1299 (3)	0.7696 (3)	0.5255 (3)	0.0384 (5)	

### Atomic displacement parameters (Å^2^)

**Table:**

	*U*^11^	*U*^22^	*U*^33^	*U*^12^	*U*^13^	*U*^23^
S1	0.0686 (4)	0.0278 (3)	0.0474 (4)	−0.0157 (3)	−0.0254 (3)	−0.0044 (2)
O1	0.0890 (15)	0.0312 (9)	0.0844 (14)	−0.0239 (9)	−0.0267 (11)	−0.0073 (9)
O2	0.0795 (14)	0.0444 (10)	0.0588 (11)	−0.0168 (9)	−0.0295 (10)	0.0051 (9)
N1	0.0442 (11)	0.0309 (9)	0.0534 (12)	−0.0102 (8)	−0.0077 (9)	−0.0062 (8)
N2	0.0450 (10)	0.0227 (8)	0.0400 (9)	−0.0076 (7)	−0.0105 (8)	−0.0084 (7)
N3	0.0478 (10)	0.0242 (8)	0.0446 (10)	−0.0109 (7)	−0.0151 (8)	−0.0078 (7)
N4	0.0678 (14)	0.0332 (10)	0.0558 (12)	−0.0205 (9)	−0.0276 (10)	−0.0047 (9)
C1	0.0448 (12)	0.0296 (10)	0.0433 (11)	−0.0090 (8)	−0.0139 (9)	−0.0120 (9)
C2	0.0468 (12)	0.0285 (10)	0.0513 (13)	−0.0119 (9)	−0.0106 (10)	−0.0158 (9)
C3	0.0352 (11)	0.0254 (9)	0.0404 (11)	−0.0070 (8)	−0.0027 (8)	−0.0088 (8)
C4	0.0462 (12)	0.0361 (11)	0.0392 (11)	−0.0105 (9)	−0.0112 (9)	−0.0109 (9)
C5	0.0481 (12)	0.0296 (10)	0.0444 (12)	−0.0100 (9)	−0.0119 (9)	−0.0152 (9)
C6	0.0347 (10)	0.0242 (9)	0.0400 (11)	−0.0063 (8)	−0.0064 (8)	−0.0119 (8)
C7	0.0404 (11)	0.0249 (9)	0.0442 (11)	−0.0068 (8)	−0.0093 (9)	−0.0124 (9)
C8	0.0745 (18)	0.0316 (11)	0.0821 (19)	−0.0198 (12)	−0.0407 (15)	−0.0044 (12)
C9	0.0481 (12)	0.0284 (10)	0.0379 (11)	−0.0111 (9)	−0.0093 (9)	−0.0108 (8)

### Geometric parameters (Å, °)

**Table:**

S1—C9	1.685 (2)	C1—H1A	0.9300
O1—N1	1.231 (3)	C2—C3	1.381 (3)
O2—N1	1.224 (3)	C2—H2B	0.9300
N1—C3	1.473 (3)	C3—C4	1.389 (3)
N2—C7	1.281 (3)	C4—C5	1.395 (3)
N2—N3	1.379 (2)	C4—H4C	0.9300
N3—C9	1.353 (3)	C5—C6	1.397 (3)
N3—H3A	0.8600	C5—H5A	0.9300
N4—C9	1.336 (3)	C6—C7	1.498 (3)
N4—H4A	0.8600	C7—C8	1.506 (3)
N4—H4B	0.8600	C8—H8A	0.9600
C1—C2	1.388 (3)	C8—H8B	0.9600
C1—C6	1.406 (3)	C8—H8C	0.9600
			
O2—N1—O1	123.1 (2)	C3—C4—H4C	121.0
O2—N1—C3	118.7 (2)	C5—C4—H4C	121.0
O1—N1—C3	118.15 (19)	C4—C5—C6	121.2 (2)
C7—N2—N3	119.08 (18)	C4—C5—H5A	119.4
C9—N3—N2	118.64 (17)	C6—C5—H5A	119.4
C9—N3—H3A	120.7	C5—C6—C1	118.60 (18)
N2—N3—H3A	120.7	C5—C6—C7	121.53 (18)
C9—N4—H4A	120.0	C1—C6—C7	119.86 (18)
C9—N4—H4B	120.0	N2—C7—C6	114.93 (18)
H4A—N4—H4B	120.0	N2—C7—C8	125.16 (19)
C2—C1—C6	121.03 (19)	C6—C7—C8	119.91 (18)
C2—C1—H1A	119.5	C7—C8—H8A	109.5
C6—C1—H1A	119.5	C7—C8—H8B	109.5
C3—C2—C1	118.5 (2)	H8A—C8—H8B	109.5
C3—C2—H2B	120.8	C7—C8—H8C	109.5
C1—C2—H2B	120.8	H8A—C8—H8C	109.5
C2—C3—C4	122.67 (19)	H8B—C8—H8C	109.5
C2—C3—N1	118.13 (19)	N4—C9—N3	117.19 (18)
C4—C3—N1	119.20 (19)	N4—C9—S1	122.63 (17)
C3—C4—C5	118.0 (2)	N3—C9—S1	120.19 (16)

### Hydrogen-bond geometry (Å, °)

**Table:**

*D*—H···*A*	*D*—H	H···*A*	*D*···*A*	*D*—H···*A*
N3—H3A···S1^i^	0.86	2.74	3.581 (2)	166
N4—H4A···O1^ii^	0.86	2.35	3.101 (3)	146
N4—H4B···O2^iii^	0.86	2.29	3.133 (3)	166

Symmetry codes: (i) −*x*, −*y*+2, −*z*+1; (ii) −*x*, −*y*, −*z*+2; (iii) *x*−1, *y*+1, *z*−1.

## References

[bb1] Bruker (1997). *SADABS*, *SMART* and *SAINT* Bruker AXS Inc., Madison, Wisconsin, USA.

[bb2] Jian, F.-F., Zhuang, R.-R., Wang, K.-F., Zhao, P.-S. & Xiao, H.-L. (2006). *Acta Cryst.* E**62**, o3198–o3199.

[bb3] Qin, Y.-Q., Ren, X.-Y., Liang, T.-L. & Jian, F.-F. (2006). *Acta Cryst.* E**62**, o5215–o5216.

[bb4] Rozwadowski, Z., Majewski, E., Dziembowska, T. & Hansen, P. E. (1999). *J. Chem. Soc. Perkin Trans. 2*, pp. 2809–2817.

[bb5] Sheldrick, G. M. (2008). *Acta Cryst.* A**64**, 112–122. 10.1107/S0108767307043930 18156677

